# Transperineal laser ablation (TPLA) with ultrasound/MRI fusion guidance in the treatment of localized radiotherapy-resistant prostate cancer

**DOI:** 10.1259/bjro.20230042

**Published:** 2023-10-18

**Authors:** Guglielmo Manenti, Marco Nezzo, Colleen Patricia Ryan, Federico Romeo Fraioli, Beatrice Carreri, Paola Elda Gigliotti, Cecilia Angeloni, Francesca Di Pietro, Martina De Angeli, Tommaso Perretta, Rolando Maria D'Angelillo, Francesco Giuseppe Garaci

**Affiliations:** 1 Department of Diagnostic Imaging and Interventional Radiology, University of Rome Tor Vergata, Rome, Italy; 2 Department of Biomedicine and Prevention, University of Rome Tor Vergata, Rome, Italy; 3 Laboratory of Neuromotor Physiology, IRCCS Fondazione Santa Lucia, Rome, Italy; 4 Radiotherapy Unit, Tor Vergata University Hospital of Rome, Rome, Italy

## Abstract

**Objective:**

The objective of this study was to assess the technical feasibility, safety, and efficacy of transperineal laser ablation (TPLA) guided by ultrasound/magnetic resonance (MR) fusion as a salvage treatment for refractory focal prostate cancer.

**Methods:**

A total of five patients who had undergone radiation therapy (RT) for prostate carcinoma and biochemical recurrence, confirmed by both prostate-specific antigen (PSA) levels and MRI (3T mpMRI), were enrolled in this study. Focal ablation was performed using a 1064 nm diode laser. Post-ablation follow-up was conducted for a duration of 18 months, which included regular PSA sampling, 3T mpMRI, and ultrasound/MR fusion-guided biopsies systematic and targeted at the site of the focal treatment.

**Results:**

The focal ablation procedure was carried out in an outpatient setting regimen with optimal clinical and biochemical outcomes. No recurrence was detected throughout the follow-up period.

**Conclusion:**

TPLA focal treatment effectively manages local recurrences of RT refractory prostate cancer without side-effects or complications. Preservation of quality of life and functional outcomes, along with *a* >70% reduction in PSA, were achieved.

**Advances in knowledge:**

Our study investigated TPLA as a salvage treatment for low-risk recurrent prostate cancer after RT, demonstrating its tolerability, feasibility, and effectiveness.

## Introduction

Prostate cancer (PCa) is the second most common neoplasm diagnosed in males worldwide.^1^ After primary treatment, between 27% and 53% of all patients develop a PSA recurrence after radical prostatectomy (RP) or radical radiotherapy (RT) ^2^. Biochemical recurrence after primary RT is defined as a PSA increase > 2 ng/mL from the nadir value reached after RT^2^. Patients affected by PCa and biochemical recurrence (BCR) after primary RT can be stratified into two prognostic groups: Low-Risk BCR, defined as biochemical recurrence-free survival > 18 months, with an ISUP grade <4; High-Risk BCR, with time to biochemical recurrence < 18 months and an ISUP grade ≥4^3^.

According to the European Association of Urology (EAU), low-risk patients (cT1-2, N0, M0, PSA <10 ng ml^−1^, life expectancy >10 years) may undergo local procedures, while high-risk patients (cT3-4, or N1, or recurrence PSA >10 ng ml^−1^, or life expectancy <10 years) have therapeutic options represented by hormonal therapy or observation.^4,5^ In patients with BCR after RT, performing a new prostate biopsy is a fundamental predictor of outcome. In PCa patients presenting PSA failure after radical RT, there is an absence of consensus regarding the optimal management of local recurrence, and therapeutic options are represented by androgen deprivation therapy or focal treatments. A recent systematic review and meta-analysis evaluated all the studies investigating the main salvage treatments in the management of locally recurrent PCa, finding no significant differences in recurrence-free survival between different techniques.^6^ Regarding hormonal therapy for PSA-recurrence after RT, the 2021 EAU guidelines for PCa recommend not offering androgen deprivation therapy to M0 patients with a PSA-doubling time >12 months.^5^

During the last few years, interest in the application of focal treatments to PC patients has grown, with the aim of achieving long-term control of cancer and reducing morbidity associated with surgery and radiation therapy. Focal laser ablation (FLA) is a safe and effective treatment for cancer lesions in different organs, due to the very thin caliber of the applicators and the high precision of the delivered energy.^7–9^ This represents a theoretical advantage since the complication rate may be limited using minimally invasive techniques. Our goal was to evaluate the safety and effectiveness of transperineal laser ablation (TPLA) as a salvage treatment for localized refractory PCa.

## Methods and materials

We enrolled five male patients over 70 years old with a diagnosis of low-risk refractory PCa. All had undergone primary treatment with either external-beam radiation therapy (EBRT) or brachytherapy. The patients were diagnosed with a single peripheral gland intracapsular lesion with a diameter ≤2 cm and a Gleason score ≤3 + 4, confirmed by prostatic ultrasound/MRI fusion-guided biopsy and systematic biopsy (>12 samples). The PSA level was <10 ng ml^−1^. Cohort data are summarized in Table 1.

**Table 1.** Patients’ data pre-TPLA

Patient	Age	PSA pre-TPLA (ng/ml)	Lesion axial diameter (mm)	PI-RR	Gleason score
1	72	5,4	10	4	3 + 4
2	81	4	15	5	3 + 3
3	71	3,9	14	5	3 + 3
4	79	4,9	18	4	3 + 4
5	76	5,1	9	4	3 + 4

PSA, prostate-specific antigen; TPLA, transperineal laser ablation.

Ethical approval for all procedures involving human participants was obtained in accordance with the ethical standards of the institutional and/or national research committee, and in compliance with the 1964 Helsinki Declaration and its later amendments. All patients provided specific informed consent.

**Figure 1.**
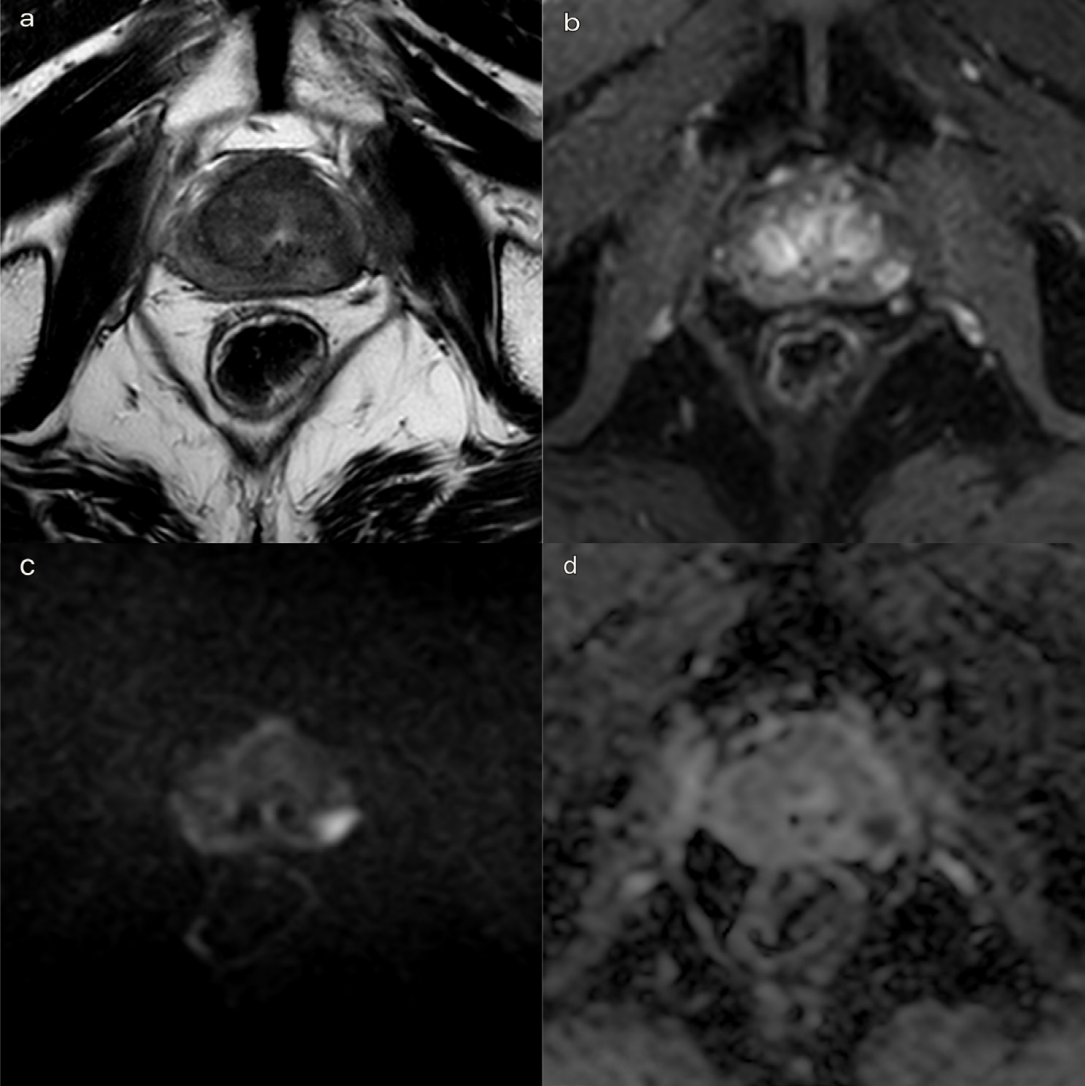
High field (3T) multiparametric MR before treatment. Focal recurrence of prostate cancer. Ultrasound/MRI-guided biopsy was performed with a Gleason score 3 + 4. (a) *T*_2_ weighted TSE sequence on axial plane: a 18 mm hypointense lesion was detected, in the peripheral zone of the left gland mid-portion. A curvilinear contact surface with the prostate capsule is demonstrated without any sign of capsule invasion. (b). DCE imaging with *T*_1_ weighted Dixon sequence with fat suppression on axial plane: early and significant focal enhancement in the peripheral zone of the left mid-portion of the gland. (c, d). DWI b-value 2500 s/mm^2^ on axial plane: focal marked hyperintensity on high *b*-value DWI sequences corresponding to a hypointensity on the ADC map (PI-RR 4). ADC, apparent diffusion coefficient; DCE, dynamic contrast-enhanced; DWI, diffusion-weighted imaging; PI-RR, prostate imaging for local recurrence reporting; TSE, turbo spin echo.

A 3T multiparametric prostate MRI (mpMRI) was performed for ultrasound/MR-guided biopsy sampling and treatment planning (Figure 1–d), following the prostate imaging for local recurrence reporting (PI-RR) score.^10^

**Figure 2.**
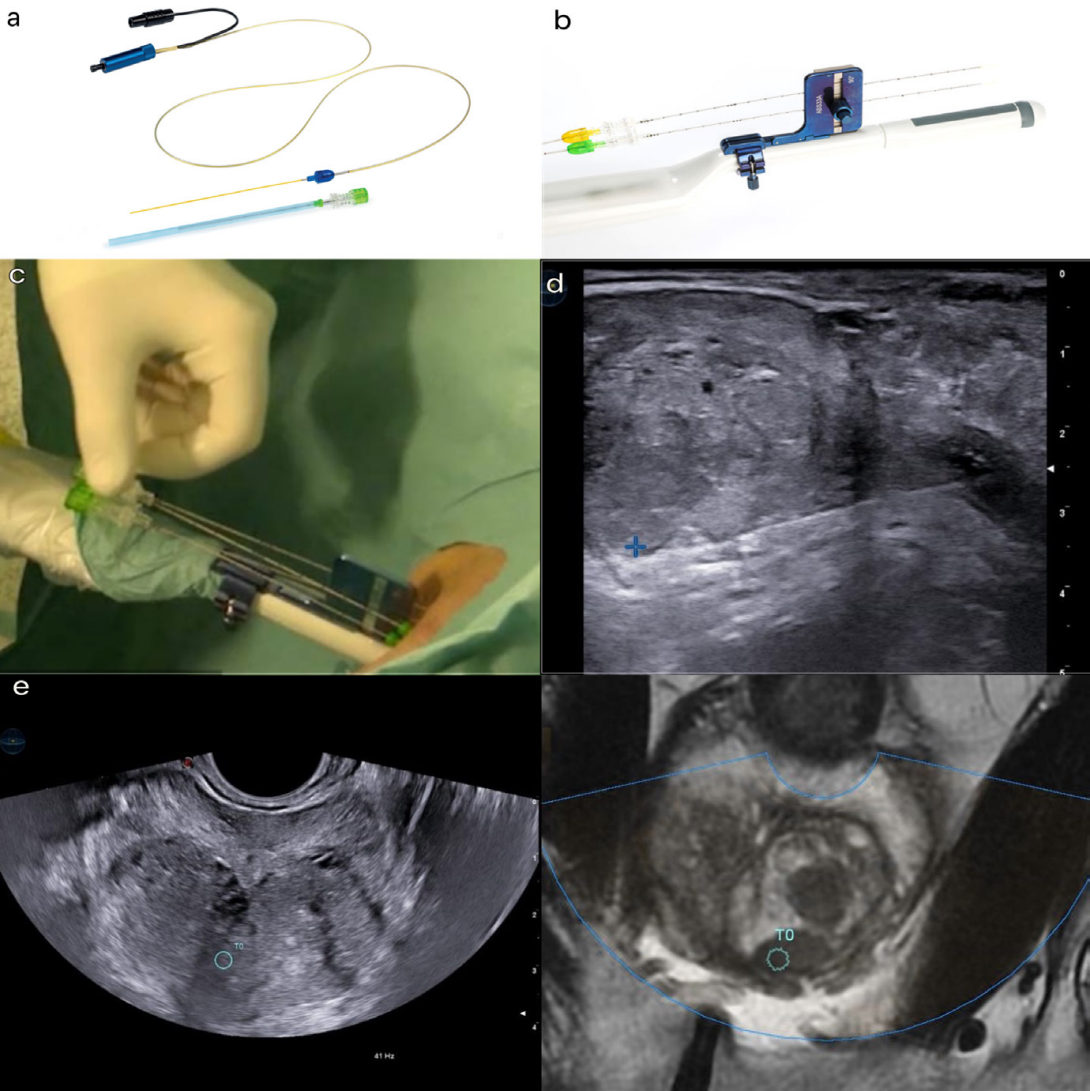
Equipment and settings. (a) A 21G trocar needle was used to accommodate the 300 micrometer flat-tip optical fiber (*Elesta SpA, Calenzano, Italy*) operating in a continuous mode with a wavelength of 1064 nm with energy setting of 1800 J at a fixed power of 3 W. The initial planning was conducted on the biplanar TRUS to ensure adequate margins of safety hence the needle tip must be at least 15 mm from the base of the bladder and 8–10 mm from the urethra and prostate capsule to ensure a safe procedure. (b). Biplanar transrectal probe (*TLC 3–13, Esaote Biomedica, Genoa, Italy*) with dedicated aligner for correct positioning and spacing of the guiding needle/fiber. Lidocaine 2% was administered to the perineum and the periprostatic region for local anesthesia. (c). A 21G Chiba needle was inserted percutaneously in the perineum under TRUS/MRI fusion imaging guidance. Energy delivered through the laser fiber passed through the needle. The needle was inserted as parallel to the longitudinal axis of the prostate as possible, with the laser fiber protruding 5–10 mm outside the tip of the needle. *Pull-back* maneuver was used to ensure adequate ablation of the indexed tumor. (d). The Soractelite EchoLaser, Elesta laser ablation system was used. A dedicated planning tool (*ESI, EchoLaser Smart Interface, Elesta SpA, Calenzano, Italy*) with simulation software allowed the user to plan the treatment and to place applicators in the prostate in a safe manner. (e). TRUS/MRI fusion images: blue circle is the target of ablation. The ellipsoid area of coagulative necrosis extends to 16-18 mm longitudinally (of which two-thirds is localized beyond the tip of the fiber and one-third behind the tip) and 10-12 mm transversely. The treatment ablation time ranges from 400 to 600 s and was concluded when the gas formation covered the entire planned ablation area and the maximum energy of 1800 J/fiber was reached.

The procedure was performed by a radiologist with over 10 years of experience in prostate interventional procedures, in an outpatient radiological intervention room. The patient was placed in the lithotomy position, and a three-way Foley 18 F catheter was inserted with continuous irrigation of saline solution. Using a dedicated system (EchoLaser SoracteLite^TM^), ultrasound/MRI fusion was used to identify the focal lesion in the prostate to be treated (Figure 2e). Local superficial anesthesia of the perineal region, followed by a transrectal prostatic block with a lidocaine solution 2% (20 ml), was performed. Under real-time ultrasound guidance, a 21G Chiba needle was inserted through the perineum into the prostate target, and a bare optic fiber (*Elesta SpA, Calenzano, Italy*) was inserted, protruding 10 mm from the tip of the needle.

The insertion of the needle was performed using a biplanar probe with a dedicated guiding system device that allowed for regular positioning and spacing of the needles (Figure 2a–d). The optic fiber was then connected to a multisource laser system operating at 1064 nm (*EchoLaser X4, ELESTA SpA, Calenzano, Italy*). A planning tool device (*ESI, EchoLaser Smart Interface, Elesta SpA, Calenzano, Italy*) was connected to the general ultrasound scanner and used for treatment planning. Ciprofloxacin 500 mg was administered as antibiotic prophylaxis. Laser administration occurred at a fixed power of 3 W, and the treatment lasted until an energy of 1800 J was reached by the fiber. The *pull-back* technique was always performed, which involved pulling back the introducer by 1 cm and performing a second illumination to increase the ablation area.

**Figure 3.**
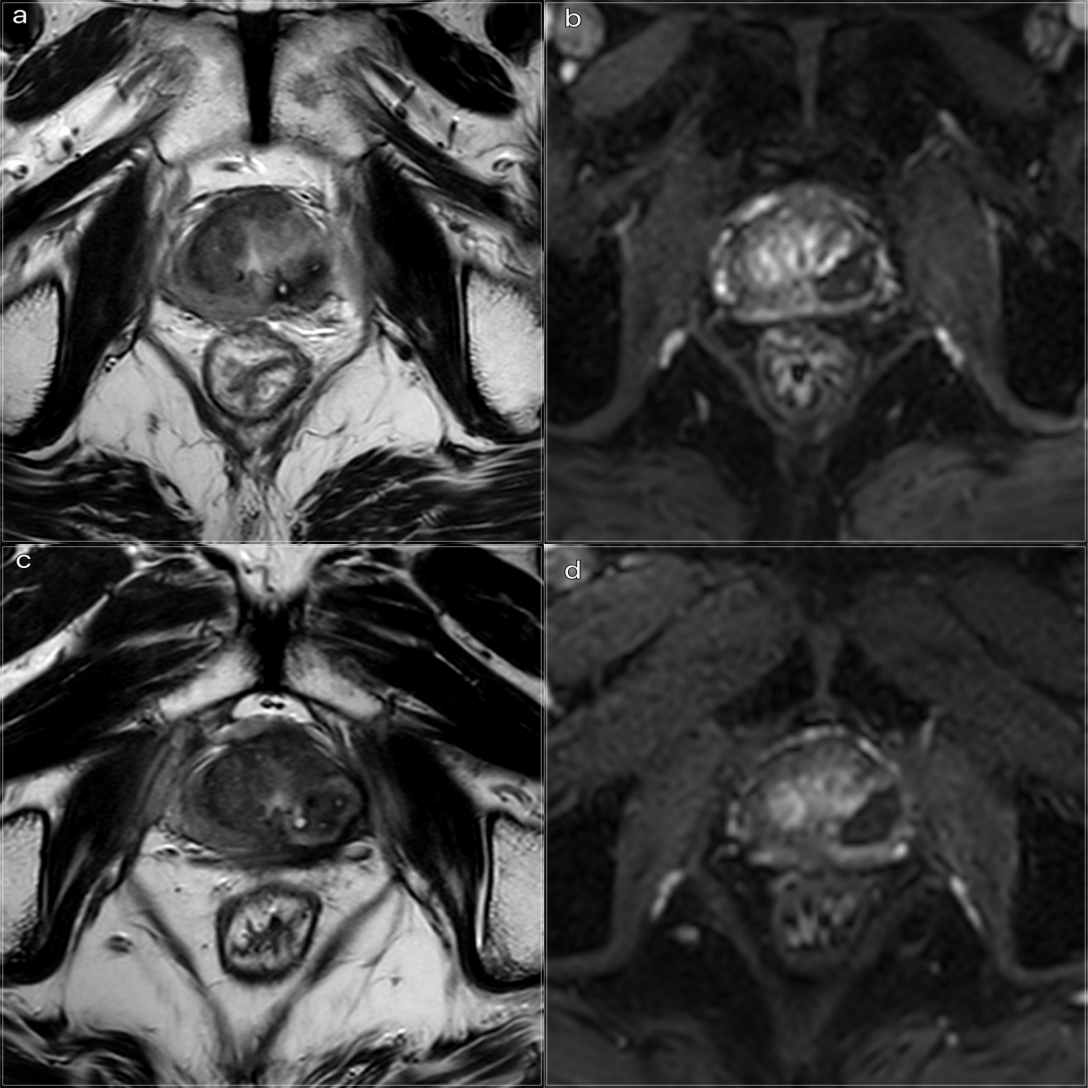
3T mpMRI performed at 1 h and 3 months after treatment respectively. (a) *T*_2_W TSE sequence on axial plane 1 h after the treatment: an elliptical ablation cavity superimposing the lesion, filled with blood derivatives and fluid as a result of tissue ablation. The hyperintense millimetric fiber tracks are visible as white dots within the cavity. (b). DCE *T*_1_W Dixon sequence 1 h after the treatment: non-enhancing cavity in the left peripheral zone. (c). *T*_2_W sequence on axial plane at 3 months follow-up: wider ablative lesion with core charring, a hyperintense thick rim of necrotic tissue with an outer hypointense thin rim of hemorrhage. Fiber tracks are still visible white spots. Bulging of the capsule and liponecrosis on the left recto-prostatic triangle. (d). DCE *T*_1_W Dixon sequence on axial plane at 3 months follow-up: unenhancing ablation cavity with a thick margin. DCE, dynamic contrast-enhanced; TSE, turbo spin echo.

After the laser ablation, all patients underwent a clinical observation period with an average time of 2 h. After 1 h, the subjects underwent a 3 T mpMRI with contrast agent (ProHance 0.5 M) to evaluate the extent of the coagulation zone (Figure 3a, b). After removal of the urinary catheter, the patient was discharged.

As proposed by Pacella et al,^11^ we followed up with patients through PSA measurements and MRI at 1 h, 3, 6, 12, and 18 months after the percutaneous treatment. The mpMRI sequences are summarized in Table 2. The volume estimation of the ablation site on MRI was calculated using a contrast-enhancement MR imaging post-processing automated segmentation software (*Philips IntelliSpace Portal 7.0 MultiModality Tumor Tracking software*).

**Table 2.** 3T multiparametric MRI protocol for patient selection and follow-up

Sequence	Plane	Time (min:s)	Repetition time (ms)	Echo time (ms)	Flip angle (°)	Number of slices	Slice thickness (mm)	Pixel size (mm)
*T*_2_ weighted turbo spin echo	Sag	2:45	3293	110	90	25	3	0.7 × 0.9
*T*_2_ weighted turbo spin echo	Ax	5:42	3000	110	90	24	3	0.6 × 0.9
Diffusion-weighted imaging^a^	Ax	6:24	3459	88	90	24	3	2.4 × 2.8
*T*_2_ weighted turbo spin echo	Cor	5:06	3000	110	90	24	3	0.5 × 0.8
*T*_1_ weighted Dixon dynamic (30 phases)	Ax	3:35	3.7	1.37	10	25	3	1.5 × 1.5
*T*_1_ weighted turbo spin echo	Ax	2:21	561	8	90	31	5	1.0 × 1.0

Ax, Axial; Cor, Coronal; Sag, Sagittal.

a b-values: 0, 1000, 1500, 2500 s/mm^2^.

Systematic and target ultrasound/MRI fusion-guided prostate biopsy was performed at 18 months.

To assess quality of life (QoL), questionnaires including the International Prostate Symptom Score (IPSS) and the 5-item version of the International Index of Erectile Function (IIEF-5) were completed at baseline and during the 18-month follow-up to investigate any procedure-related erectile dysfunction or urinary symptoms. A *t*-test for paired data was used to compare this data, with a *p*-value <0.05 considered statistically significant. Statistical analysis was performed using Stata 15 (*StataCorp 2017, College Station, TX*).

Treatment success was defined as a negative target and random biopsy, or the presence of recurrent/residual GS 3 + 3 in combination with a negative mpMRI. Residual in-field spots with GS ≥3 + 4 at TRUS/MRI-guided biopsy were defined as treatment failure, even with a negative mpMRI.^12^

## Results

Procedures were successfully completed (100% technical success) in all cases. During the clinical post-treatment observation period, no significant complications occurred, recorded according to Clavien-Dindo as Grade I.^13^ All patients were dismissed on the same day of treatment.

We evaluated the percentage change over time in PSA pre- and post-procedure and post-procedure ablation volume.

**Figure 4.**
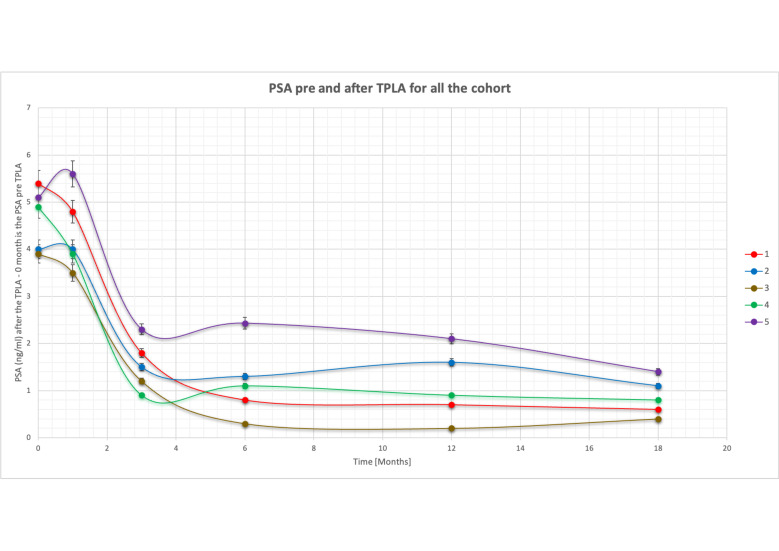
PSA before and after TPLA for all the cohort. The graph shows the trend in PSA over time (0–18 months). Time 0 stands for the pre-treatment value. A decrease over time is observed in all patients except at the first month, in which there is a slight increase. A reduction of more than 70% is observed at 18 months for all five patients. PSA, prostate-specific antigen; TPLA, transperineal laser ablation.

PSA measurement at baseline, and at 1, 3, 6, 12 and 18 months after treatment are summarized in Table 3 and Figure 4. There was a statistically relevant reduction of PSA trend at the end of follow-up (>70%) with an exception at the 1 month sample most likely related to gland ablation necrosis with a stable value compared to the baseline.

**Table 3.** PSA, MR imaging and clinical results

	Pre-procedural	1 h after treatment	1 month follow-up	3 month follow-up	6 month follow-up	12 month follow-up	18 month follow-up	% reduction at 18 months	*p*-value
PSA (ng/mL)	4,6 ± 0,7		4,4 ± 0,8	1,5 ± 0,5	1,2 ± 0,7	1,1 ± 0,8	0,9 ± 0,4	>70%	
Necrotic cavity volume (cc)		1,66 ± 0,3		4,03 ± 0,8	1,42 ± 0,7	0,71 ± 0,3	0,24 ± 0,2	>70%	
IPSS	6,8 ± 4,8						7,2 ± 3,7		0.459
IIEF-5	10,4 ± 1,9						9,8 ± 1,5		0.362

PSA, prostate-specific antigen.

After the first month, we registered a progressive decrease in time of PSA, with a decrease >70% at 18 months in all patients (Figure 4).

mpMRI performed at 1 h after treatment showed a devascularized volume up to 3x compared to the focal lesion pre-treatment 1 in all cases. Ablation cavity shape was reproducible in all the cases.

Subsequent mpMRI follow-up demonstrated the temporal evolution of treatment outcomes, including quantification of thermal damage extension (*e.g.* avascular area/volume on T1 THRIVE dynamic post-contrast acquisition), progression of the treated area/volume, and its shrinkage after the third month, without any reliable focal contrast-enhancement recurrence. MRI volume estimations at the ablation site are reported at 1 h and at 3, 6, 12, and 18 months in Table 3.

**Figure 5.**
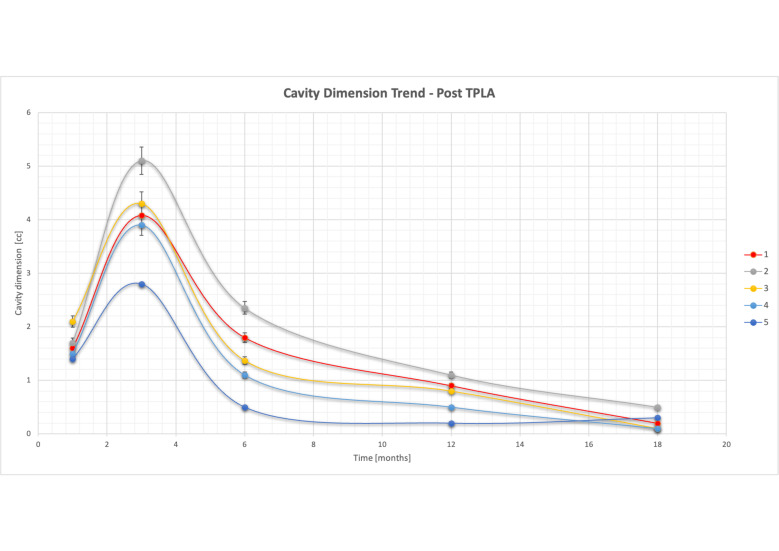
Ablation cavity dimension trend during follow-up. The graph shows a progressive decrease over time of ablation cavity volume, starting after month 3, reaching <70% of the initial value at 18 months in all five patients. TPLA, transperineal laser ablation.

**Figure 6.**
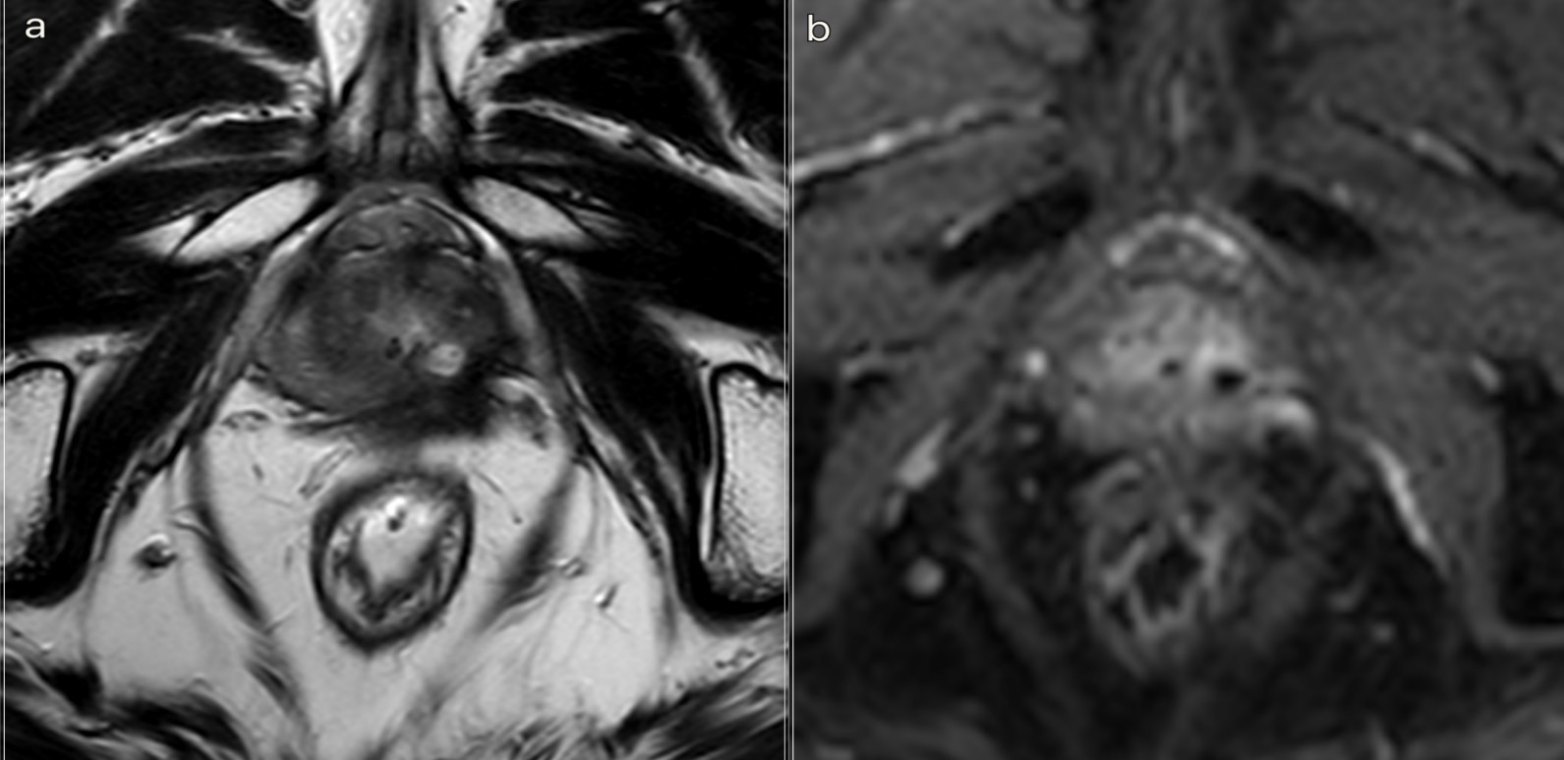
3T multiparametric MRI performed at 18 month follow-up. (a) *T*_2_W sequence: small cavity filled with fluid. A huge fibrotic scar on the posterior edge of the left gland, with an external capsule retraction. (b). DCE *T*_1_W Dixon sequence with fat suppression on axial plane: millimetric unenhancing cavity resembling a small cavity filled with proteinaceous fluid. Necrotic tissue is reabsorbed. Capsule retraction is visible. Band like left side rectoprostatic angle hypointensity on *T*_2_W imaging is the effect of mesorectum liponecrosis. Left side NVD is still visible in post-contrast MR imaging. NVD, neurovascular bundle.

We observed a progressive decrease in the ablation volume over time, with a reduction of >70% at 18 months (Figure 5). At the 18 month MRI scan, a scar replaced the ablation cavity, which was significantly reduced or completely absent (Figure 6a,b). Ultrasound/MRI fusion-guided biopsies confirmed the absence of neoplasia.

IIEF-5 and IPSS scores showed no significant difference between the pre-procedural values and the values at 18 months (IPSS *p*-value = 0.459; IIEF-5 *p*-value = 0.362, pre-procedural and final values reported in Table 3).

## Discussion

Laser ablation therapy is a minimally invasive percutaneous thermal procedure that uses a 1064 nm laser light transmitted through optical fibers for a few minutes. This treatment causes heating of the affected tissue until it is irreversibly damaged *in situ*. Focal laser ablation (FLA) treatment results in a localized increase in temperature within the treated tissue, leading to irreversible coagulation necrosis.^14^

The effectiveness of TPLA has been previously documented in studies focusing on the treatment of benign prostatic hyperplasia (BPH)^7,11,15^ and localized primary PCa.^8,12,14,16,17^

Five cases of recurrent PCa post-RT were selected and treated with focal laser ablation. mpMRI was used for initial qualitative and quantitative assessment of the lesions and for post-treatment follow-up. Additionally, ultrasound/MRI fusion-guided biopsies were performed before the treatment and at the end of the follow-up period.^18^

A sufficient safety margin for ablation was achieved for all lesions. No complications related to TPLA, other than local hematoma and/or edema, were reported.

No patients experienced statistically significant changes in urinary symptoms or erectile dysfunction during the follow-up period. The TPLA procedure preserves the urethra, bladder neck, and neurovascular bundles, as reported elsewhere.^7^

All patients had a baseline PSA level below 10 ng ml^−1^ and a Gleason score of ≤7 (3 + 4). 1 month after the procedure, there was a transient increase in PSA levels. This is a common occurrence after localized prostatic procedures and therapies, attributed to the sudden release of PSA into the bloodstream by necrotic cells, and it is considered a favorable prognostic factor.^19^ At the 18 month follow-up, we observed a reduction in PSA levels.

Due to our small sample size, we assessed the percentage change in PSA levels before and after the procedure. We observed a progressive decrease of more than 70% in PSA levels at 18 months, which is a statistically significant and promising finding (Figure 4).

To the best of our knowledge, our study is the first to investigate TPLA as a salvage treatment for low-risk local recurrent PCa after RT.

Other focal treatments for recurrent localized PCa have been studied, including cryotherapy or high-intensity focused ultrasound (HIFU), utilizing different energy modalities.^20,21^

In comparison to TPLA, cryotherapy involves the use of larger needle calibers and multiple temperature probes placed throughout the gland and between the prostate and rectum for temperature monitoring. A transurethral warming device is used to prevent urethral damage. Although the size of the ice ball can be adjusted, the treated area is usually wider than necessary, resulting in ablation of the ipsilateral neurovascular bundle.^21,22^

Salvage HIFU is a relatively new treatment option for radiation-recurrent PCa, and limited data are available regarding its safety. A recent systematic review and meta-analysis also included salvage HIFU.^3^ The adjusted pooled analysis for 5-year biochemical recurrence-free survival was 52.72% (95% CI: 42.66–62.56%). However, due to the lack of follow-up data, it is not possible to formulate any recommendations for the routine clinical use of salvage HIFU.

In our experience, TPLA has shown promising results. It is a cost-effective treatment and can be performed on an outpatient basis, for selected patients. We achieved a 100% success rate with the procedure, and there were no instances of recurrence, complications, or side-effects related to the procedure (particularly urinary or erectile side-effects) during the entire 18 month follow-up period. The transperineal route, with sparing of the urethra and rectum, helped to minimize complications. One advantage of our study is that we followed up with our patients for a period of 18 months, whereas the literature studies on the clinical application of FLA had a maximum follow-up of 1 year.^14^

mpMRI was used for lesion assessment, and TRUS/MRI fusion was utilized as a guide to enable accurate tailored focal therapy. The procedure was found to be easier when dealing with lesions located in the peripheral gland and of a larger size. The use of mpMRI as the primary follow-up imaging method resulted in high accuracy for validating the procedure.^23–27^

We conducted a qualitative and quantitative evaluation of the main imaging findings at each follow-up step.

Immediate post-treatment effects, observed at 1 h, were characterized by an elliptical hypointense ablation cavity, approximately twice the size of the original lesion. The cavity was filled with fluid and blood derivatives, and hyperintense fiber tracks were visible within the treated area. The morphology of the gland and the capsule are preserved (Figure 3a,b).

At 3 months, the main findings included the laser fiber track surrounded by a larger elliptical-shaped charred tissue cavitation, which appeared hypointense on *T*_2_ weighted images. Initial signs of coagulative necrosis could be observed at the hypointense rim of the cavity, which was filled with blood derivatives and proteinaceous fluid (Figure 3c,d).

**Figure 7.**
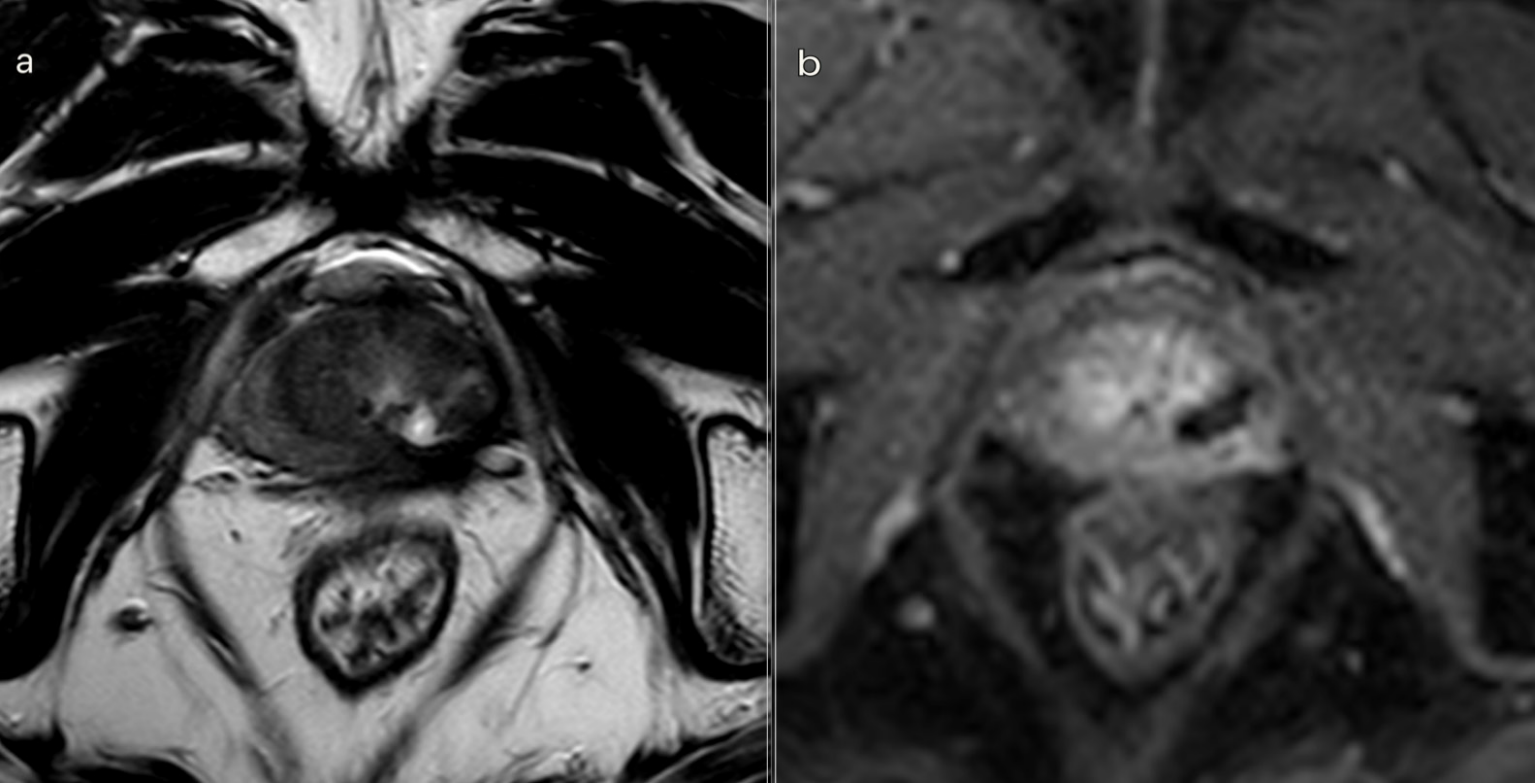
3T multiparametric MRI performed at 6 months imaging follow-up after TPLA treatment. *T*_2_W sequence on axial plane: coagulation necrosis area (hypointense on *T*_2_W imaging) is smaller because of necrotic tissue reabsorption. Figure 7b. DCE *T*_1_W Dixon sequence: non-enhancing devascularized area (hypointense on *T*_1_W + C imaging) is smaller in size compared with the third month MR-follow up. DCE, dynamic contrast-enhanced; TPLA, transperineal laser ablation.

At 6 months, within the ellipsoid cavitation, we can observe necrotic tissue. Additionally, a hypointense interface rim along the needle tracks on *T*_2_ weighted images and initial reabsorption of the cavity can be appreciated (Figure 7a,b).

At 12 and 18 months, we observed the presence of a *T*_2_ weighted hypointense scar tissue that almost completely replaced the original cavity. There were slightly visible signs of fiber tracks (Figure 6a,b).

These imaging characteristics are similar to those documented in the TPLA of BPH.^7^

Alike to the assessment of PSA levels, we also evaluated the percentage change in ablation volume over time after treatment. We observed a progressive decrease in ablation volume starting after 3 months, and by 18 months, it was less than 70% in all patients (Figure 5). It is crucial to continue the follow-up of patients to determine if the decreasing trend in PSA levels and ablation volume remains stable.

In order to establish robust statistics, it would be beneficial to increase the number of patients and potentially expand the selection criteria. Like other novel interventional techniques, TPLA requires trained operators. The operators in our study had approximately 10 years of experience, which contributed to reduced procedural time and shorter hospital stays for patients.

A limitation of our study is that we did not conduct a preliminary sample size calculation. Instead, we only included patients who met our specific criteria, such as having received whole-gland radiation therapy, having a low-risk score, no extraglandular extension, and a PSA level below 10 ng ml^−1^.

It will be important to conduct multicentric studies with a larger sample size and uniform selection criteria to confirm the efficacy of TPLA as a standard of care for the treatment of locally recurrent PCa.

## Conclusions

To the best of our knowledge, this preliminary investigation represents the first exploration of TPLA as a salvage treatment for low-risk locally recurrent PCa after RT.

The procedure itself is well-tolerated, feasible, and easily performed in an outpatient setting using local anesthesia. Patients are typically discharged within a few hours on the same day of the procedure.

Our preliminary results have demonstrated that diode laser ablation can effectively and safely treat localized recurrences of PCa over a medium-term period. Additionally, the treatment has shown favorable medium-term quality of life and functional outcomes for patients.

It is important to note that further research and larger-scale studies are needed to validate these findings and establish the long-term efficacy and safety of TPLA as a salvage treatment option for low-risk locally recurrent PCa after RT.
